# Quinidine Depresses the Transmural Electrical Heterogeneity of Transient Outward Potassium Current of the Right Ventricular Outflow Tract Free Wall

**DOI:** 10.4103/0975-3583.59979

**Published:** 2010

**Authors:** Peng Zhou, Xinchun Yang, Cuilan Li, Ying Gao, Dayi Hu

**Affiliations:** * *Heart Center, Beijing Chaoyang Hospital Affiliated Capital Medical University, Beijing, PRC, China*; † *Current affiliation: Wake Forest University School of Medicine, Winston-Salem, NC, USA*; ‡ *Department of Cardiology, Peking University People’s Hospital, Beijing PRC, China*; § *Department of Cardiology, Tongren Hospital Affiliated Capital Medical University, Being, PRC, China*

**Keywords:** arrhythmia, Brugada syndrome, idiopathic ventricular fibrillation, quinidine, transient outward potassium current

## Abstract

**Background—:**

Electrical heterogeneity of the right ventricular outflow tract (RVOT) is regarded as one of the main electrophysiological substrates for Brugada syndrome. Recently quinidine has shown efficacy in patients with Brugada syndrome due to its ability to inhibit potassium current especially 4-aminopyridine–sensitive, non-Ca^2+^ -dependent transient outward potassium current (*Ito*). However, much less is known on how extent quinidine in clinical therapeutic concentration range can inhibit this kind of electrical heterogeneity of RVOT *Ito*.

**Methods and Results—:**

Single RVOT free wall epicardial (Epi) cell, midmyocardial (M) cell and endocarcial (Endo) cells were used for whole-cell voltage clamping and *Ito* was recorded at 37°C, 0.2 Hz depolarization pulse. Evident *Ito* tranmural heterogeneity existed in RVOT free wall. Under the condition of baseline, of 10 μM quinidine perfusion 5 minutes (mins), and of 10 μM quinidine perfusion 7–10 mins, from 0 mV to 70 mV the whole transmural average *Ito* values of RVOT free wall were 10.2 pA/pF, 5.5 pA/pF and 3.5 pA/pF, respectively (between groups, *P*< 0.01). The inhibitory percentage of 10 μM quinidine at 5 mins and 7–10 mins steady-state level on the the whole *Ito* transmural heterogeneity of RVOT free wall were 46.3%±6% and 66.5%±11%, respectively.

**Conclusions—:**

There exists a robust *Ito* transmural electrical heterogeneity in RVOT free wall and quinidine in clinical therapeutic concentration can depress this kind of heterogeneity effectively.

## INTRODUCTION

It’s estimated every year there are 200,000–400,000 persons suffered from sudden cardiac death (SCD) in the USA only.^1^ SCD is the leading cause for middle-aged death and it forms a big challenge to public health all over the world.^1^ SCD most commonly occurs as a consequence of acute myocardial ischemia or develops with identifiable substrate such as scar because of previous myocardial infarction, cardiomyopathy, or hypertrophy. Despite extensive noninvasive and invasive evaluation, SCD remains unexplained without evidence of structural heart disease in about 10% of individuals surviving SCD. It is now recognized that inherited electrophysiological abnormalities, termed primary electrical diseases, are the common underlying cause of these unexplained cardiac arrests. These conditions include Wolff-Parkinson White syndrome, long QT syndrome, Brugada syndrome and catecholaminergic polymorphic ventricular tachycardia (CPVT), short QT syndrome and idiopathic ventricular fibrillation (IVF).^2^ As a kind of ion channelopathy paradigms and monogenic mutant leading to SCD, Long QT syndrome, Brugada syndrome and short QT syndrome provide us with some important keys to understand the electrophysiological mechanism of SCD.^3^–^9^ Recently, more and more experimental studies have suggested that Brugada syndrome and some IVFs originate from the right ventricular outflow tract (RVOT), and regional transmural heterogeneity is one of the main electrophysiological substrates of malignant arrhythmias caused by Brugada syndrome, short QT syndrome and some IVFs.^10^–^13^

Prominent 4-aminopyridine–sensitive, non-Ca^2+^-dependent transient outward potassium current (*Ito*) of right ventricular epicardial (Epi) cell and midmyocardial (M) cell is one of the most prominent and important manifestations of the right ventricular transmural electrical heterogeneity.^7^ The prominent *Ito* mediated action potential notch in Epi cells and M cells, but not that in endocardial (Endo) cells, create transmural voltage gradients and thus cause ST-segment elevation. Right ventricular *Ito* is an important ionic basis for repolarization “spike and dome” morphology, phase 2 “all or none” repolarization, action potential plateau heterogeneous loss and phase 2 reentry. On the basis of phase 2 reentry, ventricular tachycardia (VT) or ventricular fibrillation (VF) can be precipitated easily by a short coupling extrasystole. This is one of the most important electrophysiological mechanisms which are responsible for SCD of Brugada syndrome and some IVF victims.^7^,^12^ Because the presence of a prominent *Ito* in the RVOT is regarded as an important link to the mechanism underlying the Brugada syndrome, the important rational approach to therapy, regardless of the ionic or genetic basis for the disease, is to partially inhibit *Ito*.^14^–^17^ As a robust potassium current inhitor especially *Ito* inhibor, quinidine is effective in suppressing wedge arrhythmogenesis models of the Brugada syndrome.^12^ Clinically, although implantation of an automatic implantable cardioverter defibrillator (ICD) is the recommended first choice for most of the Brugada syndrome, short QT syndrome and IVF patients, antiarrhythmic therapy with quinidine aimed at rebalancing the currents activity during phase 1 of the right ventricular action potential is used to abort electrical storms, as an adjunct to device therapy, and as an alternative to device therapy when use of an ICD is not possible.^14^–^17^ Today, more and more evidence has shown the excellent long-term reproducibility of the electrophysiological efficacy of quinidine in patients with Brugada syndrome, short QT syndrome and some IVFs.^18^–^23^

However, limited information can be available on how effective of quinidine on restoring the *Ito* electrical homogeneity of the RVOT. In this study, we examined the *Ito* electrical heterogeneity of the RVOT free wall and the effect of clinical therapeutic concentration quinidine on this kind of *Ito* electrical heterogeneity.

## MATERIALS AND METHODS

### Prepared solutions

The following solutions were prepared as previously described by our laboratory:^24^ Ca^2+^-free buffer solution (in mmol/L): NaCl 126, KCl 5.4, MgCl_2_ 1.0, D-glucose 22, NaH_2_PO_4_ 0.9, and HEPES 5 (pH adjusted to 7.35 with NaOH); cell storage solution: Ca^2+^-free buffer solution (see above) 200 ml, BSA 0.5 g, 0.05 mmol/L CaCl_2_ 1 ml; enzymatic solution: Ca^2+^-free buffer solution (see above) 200 ml, collagenase-II 50 mg (Sigma Co., USA), proteinase-XIV 20 mg (Sigma Co., USA), BSA 200 mg, 0.05 mmol/l CaCl_2_ 1 ml; pipette solution (in mmol/L): K_2_- ASP 125, KCl 20, MgCl_2_ 1.0, Mg-ATP 5.0, HEPES 5.0, and EGTA 10.0 (pH adjusted to 7.1–7.2 with KOH); extra cellular solution (in mmol/L): NaCl 132, KCl 4.0, CaCl_2_ 2.0, MgSO_4_ 1.2, HEPES 20, and D-glucose 11.1 (pH adjusted to 7.40 with NaOH). CaCl_2_ was prepared from stock solutions just before use.

### Isolating canine right ventricular outflow tract free wall cells

The methods and protocols used in canine were approved by Capital Medical University Animal Care and Use Committee and conformed to the Guide for the Care and Use of Laboratory Animals published by the U.S. National Institutes of Health (NIH Publication 85–23, revised 1985). Cells were prepared as previously described by our laboratory and Paul G. A. Volders’experience.^24^,^25^ Briefly, 15 mongrel dogs of either sex (about 18±4 kg each) were anesthetized with pentobarbital sodium (30 mg/kg). Their hearts were excised quickly and placed into Ca^2+^-free buffer solution gassed with 95% O_2_-5% CO_2_. The right coronary artery branches, which supply blood to the free wall of the RVOT, were then cannulated and perfused with Ca^2+^-free solution at a rate of 60 ml/min. After about 15–30 mins of enzymatic solution reperfusion, samples of Epi cells (<1.5 mm from the epicardial surface), M cells (2–5 mm from the epicardial surface), and Endo cells (<1.5 mm from the endocardial surface) were dissected with a fine dermatome while carefully avoiding contamination between the three cell types. Cell samples were gently agitated, filtered, and washed, and then stored at room temperature for later use.

### *Ito* recording

**Figure 1:**
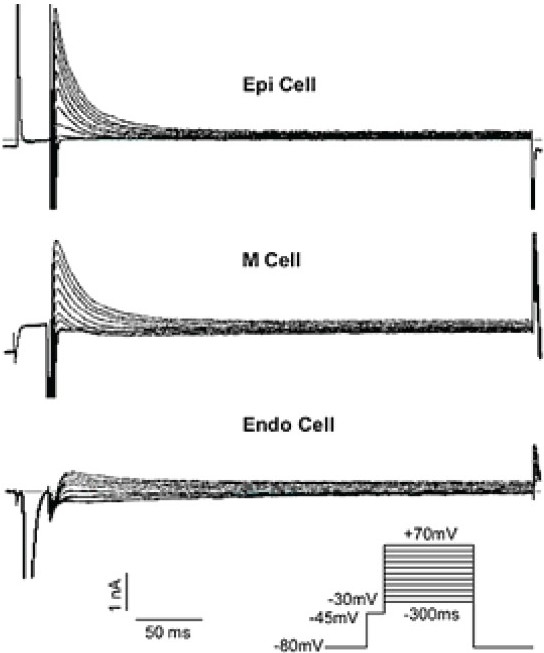
Typical *Ito* raw traces of three layer cells from RVOT free wall

**Figure 2:**
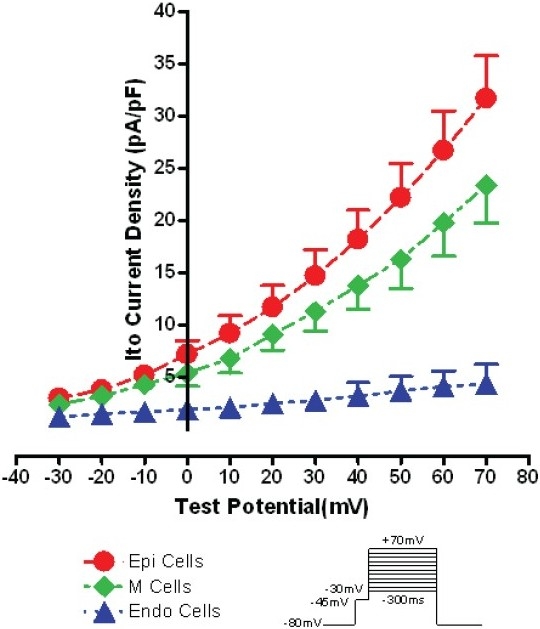
*Ito* I-V relationship of three layer cells from RVOT free wall

**Figure 3:**
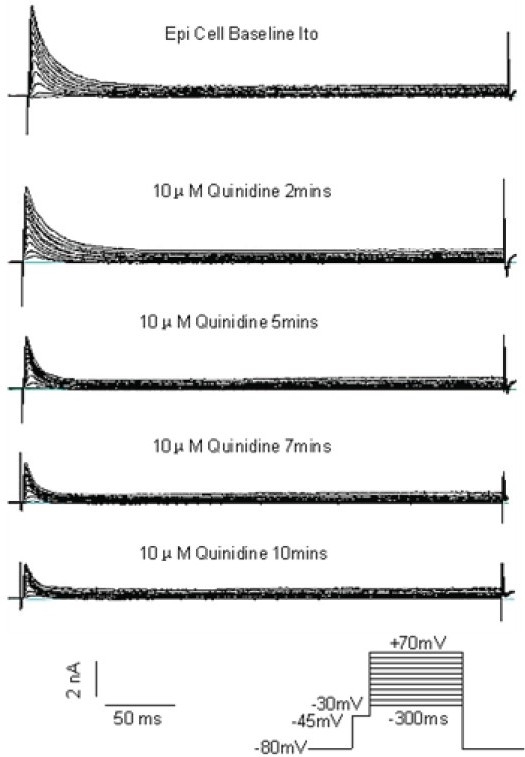
A typical example of 10 μM quinidine on the same typical Epi cell *Ito*

**Figure 4:**
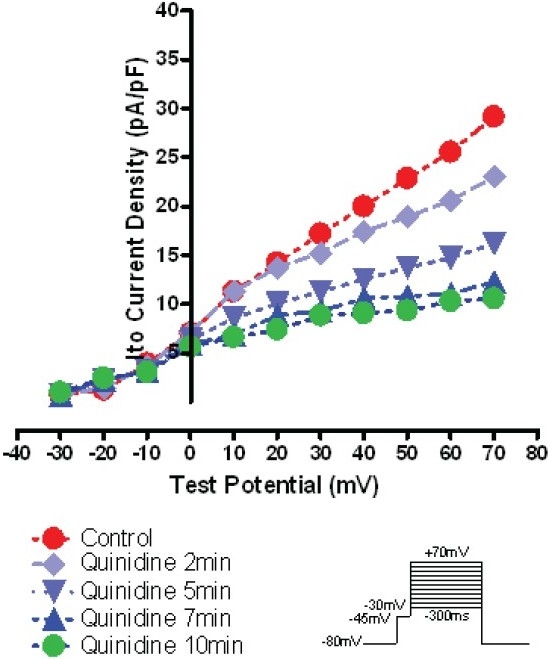
I-V relationship of a typical example of 10 μM quinidine on the same typical Epi cell *Ito*

**Figure 5:**
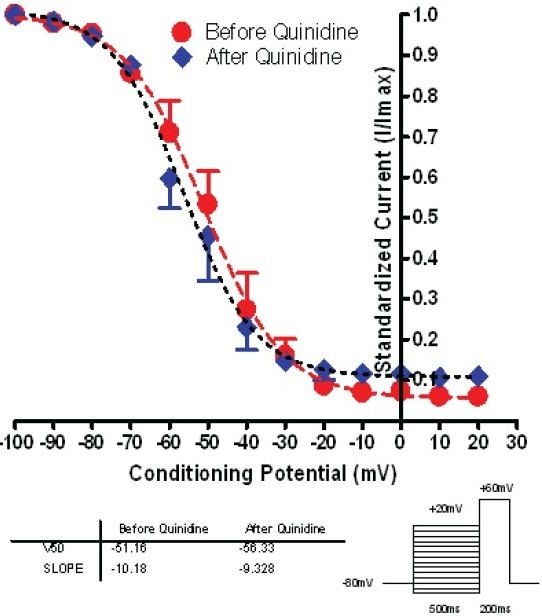
Steady-state inactivation curve of Epi cells before and after 10 μM quinidine 5mins

*Ito* was recorded using whole cell patch-clamp techniques with an EPC-9 amplifier, and controlled by Pulse+Pulsefit 8.31 software (Heka, Germany) as previously described by our laboratory.^24^ Briefly, cells were allowed to adhere to the bottom of the chamber for 3–5 mins, and then were continuously superfused with extra cellular solution at a rate of 2 ml/min. Only relaxed quiescent cells displaying prominent cross striations were used. All experiments were performed at 37°C. The resistance of all borosilicate glass pipette tips measured in extracellular solution was 2–4 MΩ when filled with pipette solution. After whole-cell configuration established, the series resistance and the slow capacitance were compensated automatically by Pulse 8.31 software to prevent large voltage errors. The activated and inactivated voltage-clamp pulse protocols are illustrated in Figures 1–5. The peak *Ito* amplitudes were measured as peak amplitudes from baseline minus steady-state values at the end of the test pulses. To analyze 4-AP-sensitive, non-calcium dependent *Ito*, it was necessary to minimize other temporarily superimposed currents. In our study, the L-type calcium current was blocked with 150 μmol/L CdCl_2_ added to the extracellular solution. T-type calcium currents, which are relatively small in canine ventricular cells, were also reduced with extracellular CdCl_2_ solution. Calcium-activated chloride currents were suppressed by blocking I_Ca_ with CdCl_2_, and by using 10 mmol/L EGTA in the pipette solution. The fast sodium current was inactivated by using a 10-ms prepulse at –45 mV (Figures 1 and 2).

### Drug

Quinidine sulfate (Beijing Municipal Chemical Plant, Beijing, China) was dissolved in the external solutions at a concentration of 10 mM and diluted to the final concentration.

### Statistical analysis

All data were expressed as mean±SEM. and analyzed with SPSS 11.5. Differences were statistically analyzed by the t test or ANOVA. *P*<0.05 was considered to be statistically significant. Figures 2, 4, 5 were produced by Graphpad Prism 4.0. Figure 6 was stimulated by SPSS (Statistical Package for the Social Science) 11.5.

## RESULTS

### Cell membrane capacitance and activation time

Cell membrane capacitance was similar in canine right ventricular RVOT free wall Epi cells (n=19), M cells (n=21), and Endo cells (n=17), with values of (156±12) pF, (159±15) pF, and (153±11) pF, respectively (between groups, *P*=NS). *Ito* in Epi, M, and Endo cells was fully activated within (3.8±1.3) ms, (3.3±1.6) ms, and (3.4±1.2) ms, respectively (between groups, *P*=NS), and reached steady-state within 100 ms (Figure 1).

### Baseline *Ito* heterogeneity between three cell types of the RVOT free wall

At different depolarizing voltage levels, *Ito* density (pA/pF) were the biggest in RVOT free wall Epi cells (n=14), followed by M cells (n=14) and, finally, Endo cells (n=12). *Ito* sometimes was absent in Endo cells (Table 1, Figure 1).

**Table 1:** Three type cells of RVOT free wall *Ito* density (pA/pF) at 0 mV and 70 mV depolarization pulses

Cell Types	Test Potential (mV)	
	0 mV	70 mV
Epi Cells (n=14)	7.3±1.3*†	31.7±4.1*†
M Cells (n=14)	5.4±1.1‡	23.4±3.6‡
Endo Cells (n=12)	1.9±0.16	4.4±1.8

Values are mean±SEM;

* *P*<0.05, Epi Cells vs. M Cells;

† *P*<0.01, Epi Cells vs. Endo Cells;

‡ *P*<0.01, M Cells vs. Endo Cells.

At 37°C, using 0.2 Hz depolarization pulses at 0 mV and at 70 mV, the average peak *Ito* density in RVOT Epi cells was bigger than that in M cells (7.3±1.3 pA/pF vs. 5.4±1.1 pA/ pF, 31.7±4.1 pA/pF vs. 23.4±3.6 pA/pF, respectively, between groups, *P*<0.05), and far greater than that in Endo cells (7.3±1.3 pA/pF vs. 1.9±0.16 pA/pF, 31.7±4.1 pA/pF vs. 4.4±1.8 pA/pF, respectively, between groups, *P*<0.01). The average peak *Ito* of M cells was significantly greater than that of Endo cells (5.4±1.2 pA/pF vs. 1.9±0.16 pA/pF, 23.4±3.6 pA/pF vs. 4.4±1.8 pA/pF, respectively, between groups, *P*<0.01). The average peak *Ito* of Endo cells was far smaller than that of Epi and of M cells (Figure 1 and 2).

### Baseline *Ito* Inactivation kinetics

The time-dependent inactivation course was best fitted with a single exponential function yielding similar time constants for Epi cells and M cells. The voltage dependence of *Ito* steadystate inactivation of Epi cells (n=11) and M cells (n=9) was well in accordance with the Boltzmann function, with half points (V1/2) of –51.2±2.6 vs. –48.4±2.6 mV (*P*=NS), respectively, and with slope factors of –10.2 ±2.5 mV vs. –9.7±2.3 mV (*P*=NS), respectively.

### Voltage Dependent Depression Effect of 10 μM quinidine on *Ito*

Figure 3 and 4 show a typical example of quinidine’s inhibitory effect on the same typical RVOT free wall epi cell. As shown in Figure 3, the depressive effect onset usually started after 10 μM quinidine superfusion about 2 mins. It would take about 7–10 mins the peak depressive effect occurred, after that, the peak depressive effect usually lasted about 10–15 mins and it was very difficult to reverse the depression effect of quinidine after washout. As shown in Figure 4, 10 μM quinidine perfusion 7–10 mins depressed *Ito* distinctly at every depolarization levels from 10 mV to 70 mV with the voltage dependent pattern. At 10mV and 70mV depolarization levels, the *Ito* depression percentage of 10 μM quinidine to RVOT free wall Epi cells were 41.3%±7% and 63.5%±11%, to M cells were 32.8%±6% and 58.6%±11%, to Endo cells were 28.2%±9% and 54.5%±13%, respectively.

Quinidine didn’t alter the voltage dependent inactivation kinetics course. Figure 5 shows the Boltzmann Sigmodal Equation fitting results for Epi cell (n=9) group before and after 10μM quinidine (V_1/2_ -51.2±2.8 mV vs. 56.3±2.5 mV, *P*=NS; Slope Factor –10.2±2.6 mV vs. –9.3±2.5 mV, *P*=NS). In M cell group, the results were consistent with Epi cells group.

### Voltage dependent depression effect of 10 μM quinidine on RVOT free wall *Ito* transmural electrical heterogeneity

Table 1, 2, and Figure 6 simulated by SPSS 11.5, show the RVOT free wall *Ito* transmural electrical heterogeneity as well as how extent 10 μM quinidine could depress this kind of electrical heterogeneity. Under baseline condition, the *Ito* density among three layer cells of RVOT free wall displayed a huge transmural electrical heterogeneity and *Ito* voltage gradient. At 0mV, the *Ito* density of Epi cell group (n=14), M cell (n=14) group and Endo myocytye (n=12) group were 7.3±1.3 pA/pF, 5.4±1.1 pA/pF and 1.9±0.16 pA/pF; At 70mV depolarization level, the *Ito* density were 31.7±4.1 pA/pF, 23.4±3.6 pA/pF and 4.4±1.8 pA/pF, respectively (Table 1). After 10 μM quinidine perfusion about 5 mins, the *Ito* density and the transmural electrical heterogeneity decreased evidently. At 0 mV, the Itodensity of Epi cell group (n=8), M cell group (n=7) and Endo cell group (n=7) were decreased to 5.5±1.1 pA/pF, 4.1±0.73 pA/pF and 1.4±0.13 pA/pF; At 70mV depolarization level, the *Ito* density were decreased to 17.4±2.4 pA/pF, 12.9±2.2 pA/pF and 3.2±1.4 pA/pF, respectively (Table 2). After 7–10 mins when the depression effect of 10 μM quinidine reached steadystate, the *Ito* density and the transmural electrical heterogeneity further decreased. At 0 mV, the *Ito* density of Epi cell group (n=7), M cell group (n=7) and Endo cell group (n=5) were decreased to 4.4±0.87 pA/pF, 3.2±0.69 pA/pF and 0.87±0.1 pA/pF, respectively; At 70 mV depolarization level, the *Ito* density were decreased to 13.3±1.9 pA/pF, 9.8±1.7 pA/pF and 2.4±1.1 pA/pF, respectively (Table 2).

**Table 2:** After perfusion 5mins and 7–10mins, the voltage dependent inhibItory effect of 10 μM quinidine on RVOT free wall *Ito* (pA/pF)

Cell Types	0mV			70mV		

	Baseline	5mins	7–10mins	Baseline	5mins	7–10mins
Epi Cells	7.3±1.3	5.5±1.1*	4.4±0.87**	31.7±4.1	17.4±2.4†	13.3±1.9††
M Cells	5.4±1.1	4.1±0.73‡	3.2±0.69‡‡	23.4±3.6	12.9±2.2§	9.8±1.7§§
Endo Cells	1.9±0.16	1.4±0.13¶	0.87±0.1¶¶	4.4±1.8	3.2±1.4#	2.4±1.1##

Values are mean±SEM;

* *P*<0.05, 10 μM quinidine 5 mins vs. Baseline;

† *P*<0.01, quinidine 5 mins vs. Baseline;

‡ *P*<0.05, 10 μM quinidine 5 mins vs. Baseline;

§ *P*<0.01, quinidine 5 mins vs. Baseline;

# *P*<0.01, quinidine 5 mins vs. Baseline;

¶ *P*<0.05, 10 μM quinidine 5 mins vs. Baseline;

** *P*<0.01, quinidine 7–10 mins vs. Baseline;

†† *P*<0.01, quinidine 7–10 mins vs. Baseline.

‡‡ *P*<0.01, quinidine 7–10 mins vs. Baseline;

§§ *P*<0.01, quinidine 7–10 mins vs. Baseline.

¶¶ *P*<0.01, quinidine 7–10 mins vs. Baseline;

## *P*<0.01, quinidine 7–10 mins vs. Baseline.

At baseline condition, 10 μM quinidine perfusion 5 mins and 7–10 mins, from 0 mV to 70 mV the whole transmural average *Ito* values (which were expressed by green planes in (Figure 6A, B, C) of RVOT free wall were 10.2 pA/pF, 5.5 pA/pF and 3.5 pA/pF, respectively (between groups, *P*<0.01). The inhibitory percentage of 10 μM quinidine at 5 mins and 7–10 mins steadystate level on the the whole *Ito* transmural heterogeneity of RVOT free wall were 46.3%±6% and 66.5%±11%, respectively.

**Figure 6A:**
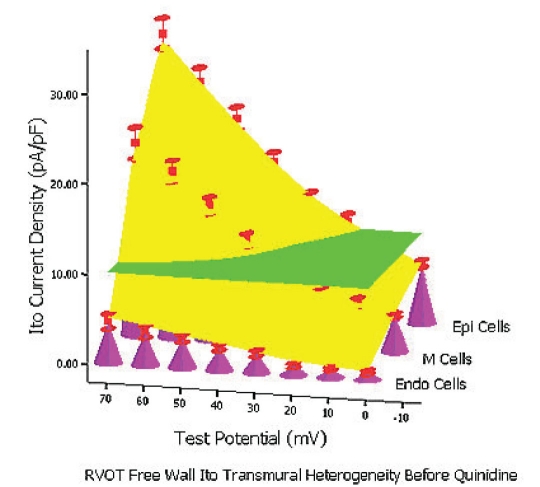
Baseline RVOT Free Wall *Ito* Transmural Electrical Heterogeneity 3-Dimension Figure Simulated by SPSS 11.5: X axis stands for test potential; Y axis stands for *Ito* density; Z axis stands for three layer cells; The height of pink cones stands for *Ito* density of each cell types at different test potential; The green plane stands for the whole transmural average *Ito* values of RVOT free wall; The yellow bent smooth plane stands for the whole transmural *Ito* gradient of RVOT free wall.

**Figure 6B:**
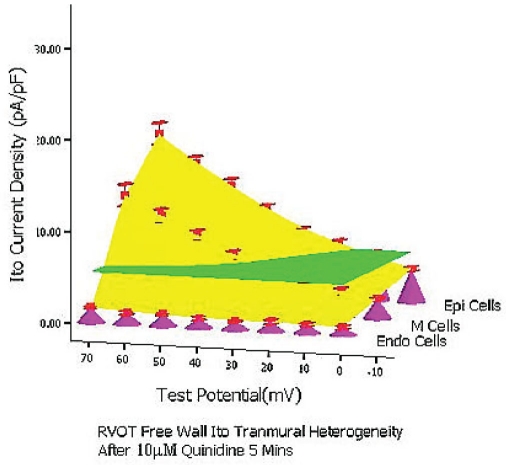
After 10 μM quinidine perfusion 5mins

**Figure 6C:**
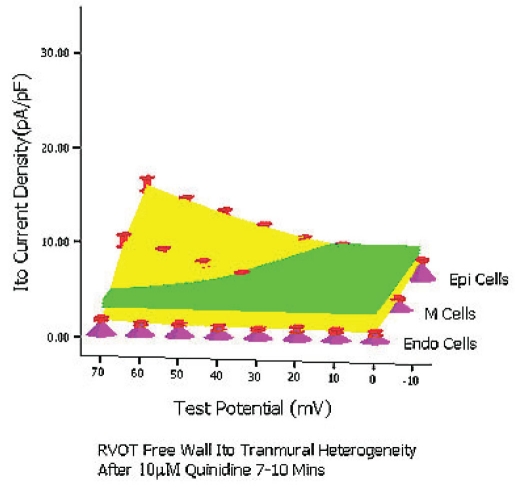
After 10 μM quinidine perfusion 7–10mins

## DISCUSSION

The data above clearly demonstrated that there exists strong transmural *Ito* electrical heterogeneity in RVOT free wall. Quinidine at the concentration of 10 μM, which was in clinical therapeutic range, possesses the ability to depress the strong transmural *Ito* electrical heterogeneity of RVOT free wall effectively.

### Electrical heterogeneity of three types of canine right ventricular cells

Using arterially perfused wedges of canine right ventricle (RV), by simultaneously recording transmembrane action potentials from 2 epicardial and 1 endocardial sites, together with unipolar electrograms and a transmural Electrocardiograph (ECG), Yan et al. demonstrated that depression or loss of the action potential dome in RV epicardium creates a transmural voltage gradient that may be responsible for the ST-segment elevation observed in the Brugada syndrome and other syndromes exhibiting similar ECG manifestations. Their results also demonstrated that extrasystolic activity due to phase 2 reentry can arise in the intact wall of the canine RV and serve as the trigger for VT/VF. Their data also pointed to *Ito* blocker (4-aminopyridine, quinidine) as an effective pharmacological treatment.^12^ However, for the RVOT free wall, which was regarded as the seedbeds of Brugada syndrome and some IVF, the information on trnasmural electrical heterogeneity, especially *Ito* trnasmural electrical heterogeneity of RVOT free wall was very limited. Our data above clearly indicated that there exist marked *Ito* electrical heterogeneity and transmural wall voltage gradients between Epi cells and Endo cells, and between M cells and Endo cells in the canine RVOT free wall. So, our results not only confirm the existence of potent *Ito* electrical heterogeneity in the RVOT free wall, but also provide further convincing quantitative evidence supporting the hypothesized mechanism for J wave formation, J point elevation, and ST-segment elevation on ECG of right precordial leads.

### Clinical relevance

The electrical heterogeneity of the right ventricle has a close relationship with certain right ventricular malignant arrhythmias found in patients with Brugada syndrome and some IVFs.

Recently, more and more studies have verified in most rodents, canines, and humans, there is obvious *Ito* electrical heterogeneity in the ventricles during repolarization (phase 1). The different *Ito* distributions and transmural voltage gradients are among the most prominent and most important manifestations of right ventricular electrical heterogeneity, and one of the important ionic bases for the “spike and dome” action potential differences between the three cell types, and for J point elevation, J wave formation and ST-segment elevation, action potential asymmetrical loss, “all or none” repolarization, and reentry into phase 2. VT or VF induced by phase 2 reentries, as a result of powerful transmural *Ito* electrical heterogeneity in the right ventricle during repolarization (phase 1), may be one of the main electrophysiological mechanisms for sudden cardiac death in patients with Brugada syndrome and other conditions^3^–^8^,^10^,^12^ although, there are still some controversies.^26^

Under the supporting of the electrical heterogeneity and the repolarization disorder theory above, recently quinidine, as an adjunct to ICD therapy, has been used in treatment of IVF, Brugada syndrome and short QT syndrome successfully. More and more papers reported the profit of antiarrhythmic therapy with quinidine as an adjunct to ICD therapy to abort electrical storms, or the profit of quinidine as an alternative to device therapy when use of an ICD is not possible.^14^,^15^,^17^–^23^ However, limited information can be available on the *Ito* transmural electrical heterogeneity of RVOT free wall, one important seedbed for some clinical malignant arrhythmia and much less is known how extent quinidine in clinical therapeutic concentration range can depress the *Ito* transmural electrical heterogeneity coming from RVOT free wall. So, our results primarily answered the critical problems above and provided a further evidence for quinidine in treatment of some IVF, Brugada syndrome and short QT syndrome.

Based on the results of our study, we can conclude that there exists a robust *Ito* transmural electrical heterogeneity in RVOT free wall and quinidine in clinical therapeutic concentration can depress this kind of heterogeneity effectively.

## ABBREVIATIONS

Sudden Cardiac Death (SCD), Catecholaminergic Polymorphic Ventricular Tachycardia (CPVT), Idiopathic Ventricular Fibrillation (IVF), Right Ventricular Outflow Tract (RVOT), Transient Outward Potassium Current (*Ito*), Epicardial (Epi), Midmyocardial (M), Endocardial (Endo), Ventricular Tachycardia (VT), Ventricular Fibrillation (VF), Implantable Cardioverter Defibrillator (ICD).

## References

[1] Zipes DP, Camm AJ, Borggrefe M, Buxton AE, Chaitman B, Fromer M, Gregoratos G, Klein G, Moss AJ, Myerburg RJ, Priori SG, Quinones MA, Roden DM, Silka MJ, Tracy C (2006). ACC/AHA/ESC 2006 guidelines for management of patients with ventricular arrhythmias and the prevention of sudden cardiac death—executive summary: a report of the American College of Cardiology/American Heart Association Task Force and the European Society of Cardiology Committee for Practice Guidelines (Writing Committee to Develop Guidelines for Management of Patients With Ventricular Arrhythmias and the Prevention of Sudden Cardiac Death). *Circulation*.

[2] Mark Estes N.A (2005). III. Sudden Cardiac Arrest From Primary Electrical Diseases. *Circulation*.

[3] Shah M, Akar FG, Tomaselli GF (2005). Molecular Basis of Arrhythmias. *Circulation*.

[4] Rossenbacker T, Priori SG (2007). The Brugada syndrome. *Current Opinion in Cardiology*.

[5] Alings M, Wilde A (1999). “Brugada” Syndrome Clinical Data and Suggested Pathophysiological Mechanism. *Circulation*.

[6] Gussak I, Antzelebitch C, Bjerregaard P, Towbin JA (1999). The Brugada Syndrome: Clinical, Electrophysiologic and Genetic Aspects. *J Am Coll Cardiol*.

[7] Antzelevitch C (2007). Heterogeneity and cardiac arrhythmias: An overview. *Heart Rhythm*.

[8] Benito B, Brugada R, Brugada J, Brugada P (2008). Brugada Syndrome. *Progress in Cardiovascular Diseases*.

[9] Giustetto C, Monte FD, Wolpert C, Borggrefe M (2006). Short QT syndrome: clinical findings and diagnostic–therapeutic implications. *European Heart Journal*.

[10] Nagase S, Kusano KF, Morita H, Nishii N (2008). Longer Repolarization in the Epicardium at the Right Ventricular Outflow Tract Causes Type 1 Electrocardiogram in Patients With Brugada Syndrome. *. J Am Coll Cardio*.

[11] Patel C, Antzelevitch C (2008). Cellular basis for arrhythmogenesis in an experimental model of the SQT1 form of the short QT syndrome. *Heart Rhythm*.

[12] Yan GX, Antzelevitch C (1999). Cellular basis for the Brugada syndrome and other mechanisms of arrhythmogenesis associated with ST-segment elevation. *Circulation*.

[13] Extramiana F, Antzelevitch C (2004). Amplified transmural dispersion of repolarization as the basis for arrhythmogenesis in a canine ventricularwedge model of short-QT syndrome. *Circulation*.

[14] Rquez MFMA, Salica G, Hermosillo AG, Pasteli NG (2007). Ionic Basis of Pharmacological Therapy in Brugada Syndrome. *. J Cardiovasc Electrophysiol*.

[15] Yang FL, Hanon S, Lam P, Schweitzer P (2009). Quinidine Revisited. *The American Journal of Medicine*.

[16] Antzelevitch C, Brugada P, Borggrefe M, Brugada J (2005). Brugada Syndrome: Report of the Second Consensus Conference: Endorsed by the Heart Rhythm Society and the European Heart Rhythm Association. *Circulation*.

[17] Antzelevitch C, Fish JM (2006). Therapy for the Brugada Syndrome. *Handb Exp Pharmacol*.

[18] Calloe1 K, Cordeiro JM, Di Diego JM, Hansen RS (2009). A transient outward potassium current activator recapitulates the electrocardiographic manifestations of Brugada syndrome. *Cardiovascular Research*.

[19] Belhassen B, Glick A, Viskin S (2009). Excellent Long-Term Reproducibility of the Electrophysiologic Efficacy of Quinidine in Patients with Idiopathic Ventricular Fibrillation and Brugada Syndrome. *PACE*.

[20] Viskin S, Wilde AAM, Tan HL, Antzelevitch C, Shimizu W (2009). Empiric quinidine therapy for asymptomatic Brugada syndrome: Time for a prospective registry. *Heart Rhythm*.

[21] Milberg P, Tegelkamp R, Osada N, Schimpf R (2007). Reduction of Dispersion of Repolarization and Prolongation of Postrepolarization Refractoriness Explain the Antiarrhythmic Effects of Quinidine in a Model of Short QT Syndrome. *Journal of Cardiovascular Electrophysiology*.

[22] Kyriazis K, Bahlmann E, Hendrik van der Schalk, Kuck KH (2009). Electrical storm in Brugada syndrome successfully treated with orciprenaline; effect of low-dose quinidine on the electrocardiogram. *Europace*.

[23] Elizaberth S, Kaufman N (2007). Quinidine in Short QT Syndrome:An Old Drug for a New Disease. *J Cardiovasc Electrophysiol*.

[24] Yang XC, Zhou P, Li CL (2004). Electrical heterogeneity of canine right ventricular transient outward potassium currents. *Chinese Medical Journal*.

[25] Volders PGA, Sipido KR, Carmeliet E, Spätjens RLHMG (1999). Repolarizing K1 Currents *Ito* and IKs Are Larger in Right Than Left Canine Ventricular Midmyocardium. *Circulation*.

[26] Boussy T, Sarkozy A, Chierchia GB, Richter S (2007). The Brugada Syndrome: Facts and Controversies. *Herz*.

